# Corrigendum to “ISPAD Clinical Practice Consensus Guidelines 2022: Type 2 Diabetes in Children and Adolescents”

**DOI:** 10.1155/pedi/9814065

**Published:** 2025-08-21

A. S. Shah, P. S. Zeitler, J. Wong, et al., “ISPAD Clinical Practice Consensus Guidelines 2022: Type 2 Diabetes in Children and Adolescents,” *Pediatric Diabetes* 23, no. 7 (2022): 872–902, https://doi.org/10.1111/pedi.13409.

In the article titled “ISPAD Clinical Practice Consensus Guidelines 2022: Type 2 Diabetes in Children and Adolescents,” there was an error in Section 3.2.1. This error is shown below:

“[In other countries, the proportion of youth-onset T2D is reported to be 66% in Indigenous Australians 21 and 68.6% in China.]”

should read

“[In other countries, the proportion of youth-onset T2D is reported to be 66% in Indigenous Australians and 7.4% in China.]”

All authors agree with the abovementioned changes.

We apologize for this error.

